# Comparison of the osteogenic potential of mesenchymal stem cells from the bone marrow and adipose tissue of young dogs

**DOI:** 10.1186/s12917-014-0190-y

**Published:** 2014-09-02

**Authors:** Endrigo GL Alves, Rogéria Serakides, Jankerle N Boeloni, Isabel R Rosado, Natália M Ocarino, Humberto P Oliveira, Alfredo M Góes, Cleuza MF Rezende

**Affiliations:** 1 Curso de Medicina Veterinária da Universidade de Uberaba (UNIUBE), Uberaba, Brazil; 2 Núcleo de Células Tronco e Terapia Celular Animal, NCT-TCA Departamento de Clínica e Cirurgia, Escola de veterinária da Universidade Federal de Minas Gerais, Belo Horizonte, Brazil; 3 Departamento de Bioquímica e Imunologia do Instituto de Ciências Biológicas da Universidade Federal de Minas Gerais, Belo Horizonte, Brazil

**Keywords:** Mesenchymal stem cells, Osteogenic differentiation, Dogs

## Abstract

**Background:**

The aim of the present study was to compare the osteogenic potential of mesenchymal stem cells extracted from the bone marrow (BM-MSCs) and adipose tissue (AD-MSCs) of young dogs. The following parameters were assessed: dimethyl thiazolyl diphenyl tetrazolium (MTT) conversion, alkaline phosphatase (ALP) activity, collagen and mineralised matrix synthesis, and the expressions of osterix, bone sialoprotein (BSP), and osteocalcin (OC).

**Results:**

MTT conversion was greater in BM-MSCs compared to AD-MSCs after 14 and 21 days of differentiation; ALP activity was greater in differentiated AD-MSCs on day 7; collagen synthesis was greater in BM-MSCs on days 14 and 21; the percentage of mineralized area per field was greater in BM-MSCs compared to AD-MSCs; osterix expression was greater in BM-MSCs in days 14 and 21, and BSP and OC expression levels were greater in BM-MSCs at all the investigation time-points.

**Conclusions:**

It was concluded that the osteogenic potential was greater in BM-MSCs than AD-MSCs when extracted from young dogs.

## Background

The aim of cell therapy research is to produce successful cell cultures that can be used in the treatment of various disorders. However, it is a subject of on-going research, and many questions remain unanswered. The available data show that there are various populations of mesenchymal stem cells (MSCs) that exhibit variable patterns of proliferation and differentiation within the same individual [1]. These populations are unevenly distributed in the tissues, and the tissue site determines the proliferative and differentiation potential of MSCs isolated from the same individual [2]. Some studies have shown that the osteogenic potential of MSCs may vary as a function of tissue type [3],[4], collection site [5], animal species [6], age [7],[8], state of health [9], and performance of physical activity on a daily basis [7],[10].

*In vitro* comparison of the osteogenic potential of various MSC sources allows for the selection of the most promising MSCs for bone regeneration therapy. Bone marrow [11] and adipose tissue [12] are considered to be the main sources of MSCs due to the presence of large numbers of cells and the simplicity of collection and isolation. However, the best source of MSCs for bone regeneration therapy remains unknown [2]. Few studies have compared bone marrow and adipose tissue MSCs in dogs. One such study compared the *in vivo* osteogenic potential of MSCs from bone marrow, adipose tissue, and umbilical cord blood in treating bone defects in dogs [4]. However, no study has yet compared the *in vitro* osteogenic potential of MSCs from the bone marrow and adipose tissue of the same animal.

Therefore, the aim of the present study was to perform a quantitative assessment of the osteogenic potential of bone marrow (BM-MSCs) and adipose tissue (AD-MSCs) MSCs from young dogs, in order to identify the best source of MSCs for bone regeneration therapy in dogs. The hypothesis is that the BM-MSCs present higher osteogenic potential than the AD-MSCs, since in the organism these cell these cells differentiate naturally into osteoblasts.

## Results

### Cell phenotype characterization

Both BM-MSCs and AD-MSCs exhibited an elongated fusiform shape. Phenotype characterization found high expression of the stem cell markers CD90 (80.04% ± 1.96) and CD29 (96.00% ± 3.00) in BM-MSCs and CD90 (60.94% ± 4.45) and CD29 (77.08% ± 2.88) in AD-MSCs. The expression of haematopoietic cell markers was low: CD45 (1.45% ± 0.60) and CD34 (1.53% ± 0.39) in BM-MSCs and CD45 (1.54% ± 0.46) and CD34 (0.88% ± 0.30) in AD-MSCs.

### Conversion of MTT into formazan and alkaline phosphatase activity

**Figure 1:**
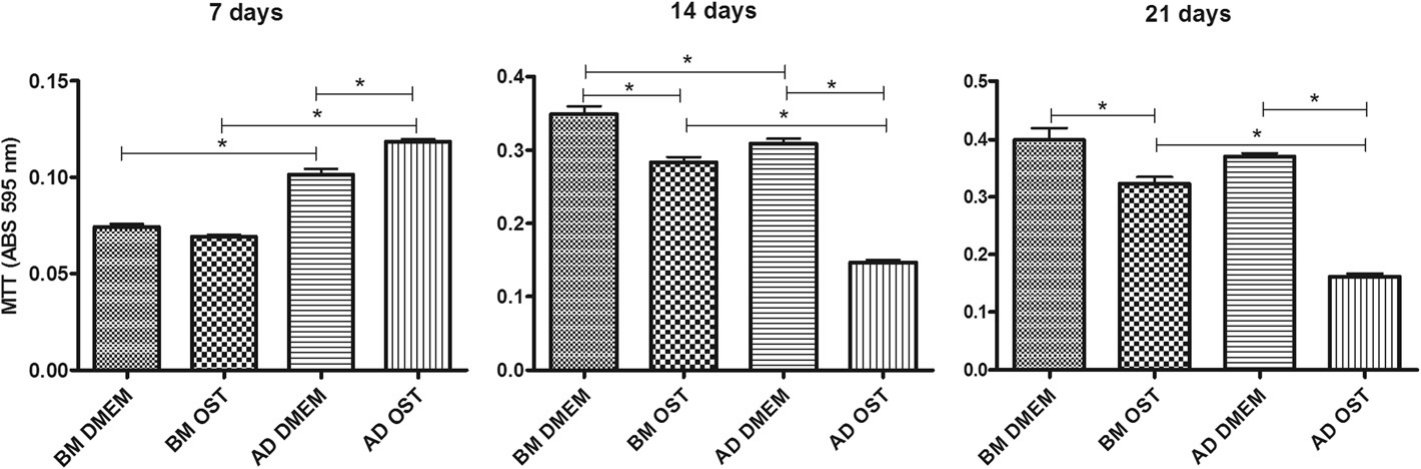
Mean and standard deviation values of MTT conversion into formazan crystals in canine BM-MSC and AD-MSC cultures in DMEM (basal medium) or osteogenic medium on days 7, 14, and 21 *(P < 0.05).

All of the groups exhibited an increased ability to convert MTT into formazan crystals throughout the culture period independent of the medium used. MSCs cultured in basal medium exhibited greater MTT conversion compared to those cultured in osteogenic medium on days 14 and 21. When subjected to osteogenic differentiation, BM-MSCs exhibited greater MTT conversion compared to AD-MSCs under the same culture conditions on days 14 and 21 (Figure 1).

**Figure 2:**
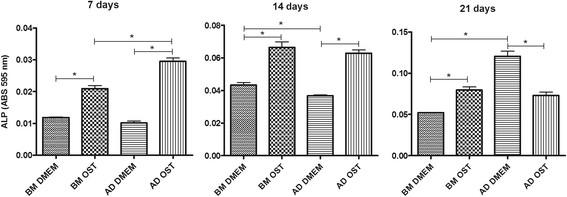
Mean and standard deviation values of alkaline phosphatase activity in canine BM-MSC and AD-MSC cultures in DMEM (basal medium) or osteogenic medium on days 7, 14, and 21 *(P < 0.05).

Except for AD-MSCs on day 21, all of the cells exhibited greater ALP activity when cultured in osteogenic medium. Relative to the cultures in osteogenic medium, AD-MSCs exhibited greater ALP activity on day 7 compared to BM-MSCs. This increased activity was not found at the later time-points of assessment (days 14 and 21) (Figure 2).

### Collagen synthesis, percentage of cells, and mineralised matrices

**Figure 3:**
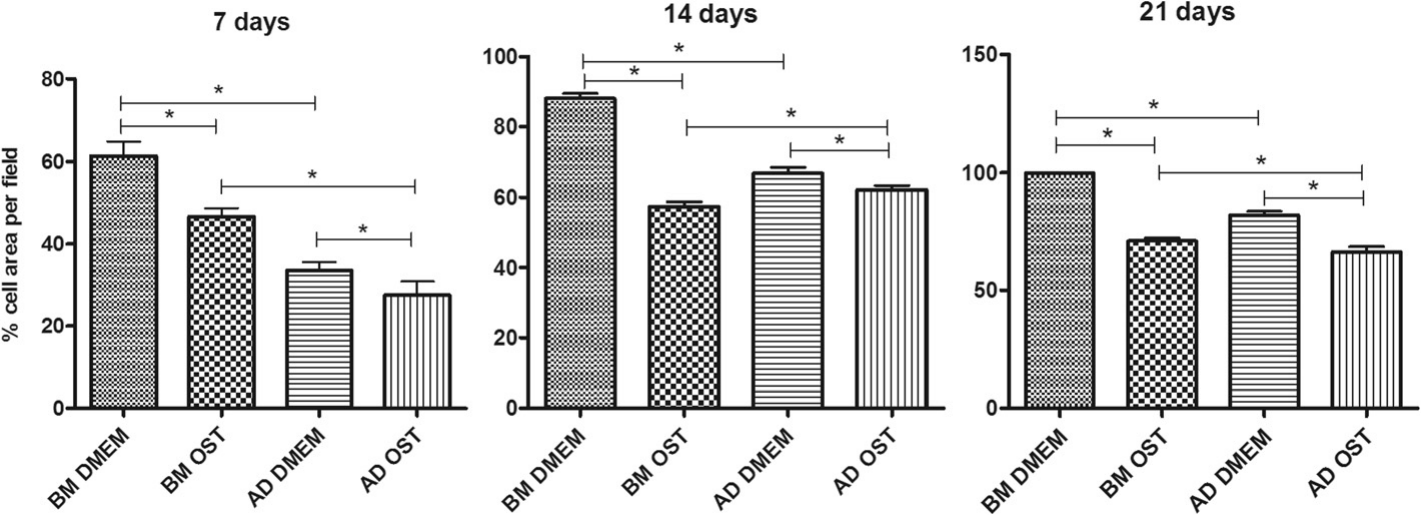
Mean and standard deviation of the percentage of cells per field in canine BM-MSC and AD-MSC cultures in DMEM (basal medium) or osteogenic medium on days 7, 14, and 21 *(P < 0.05).

All of the groups exhibited an increase in the percentage of cells per field throughout the culture period regardless of the medium used. MSCs in basal medium systematically exhibited a higher number of cells per field compared to those subjected to osteogenic differentiation, although the initial number of cells was the same. The number of cells per field corresponding to undifferentiated BM-MSCs was higher compared to that of AD-MSCs under the same culture conditions at all the investigated time-points. When cultured in osteogenic medium, the number of cells per field corresponding to BM-MSCs was higher than that of AD-MSCs under the same culture conditions on days 7 and 21 (Figure 3).

**Figure 4:**
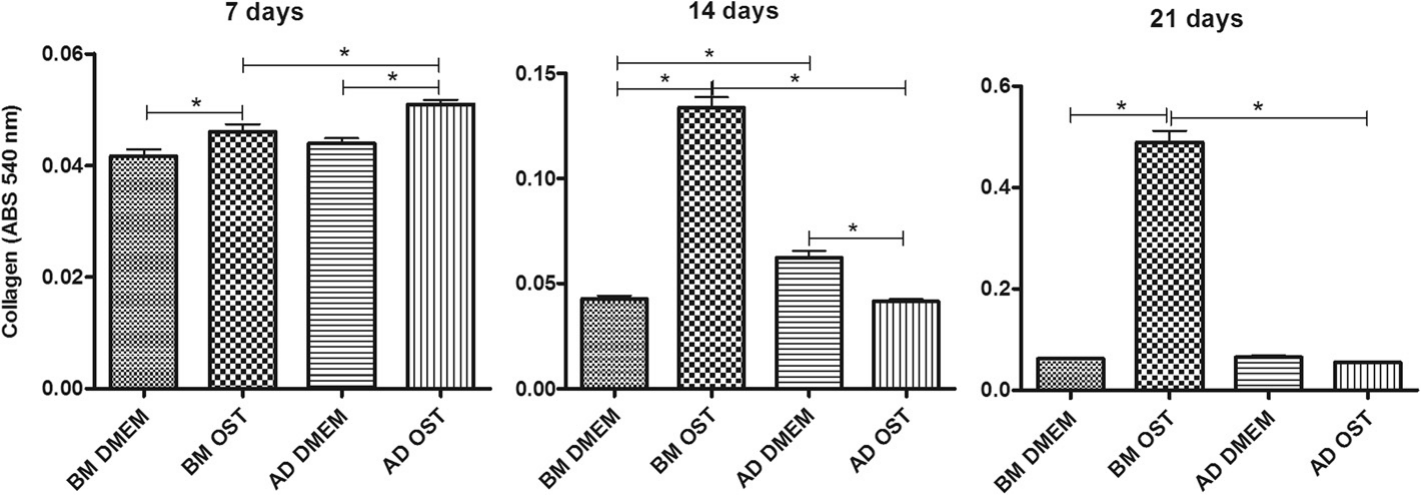
Mean and standard deviation values of collagen content in canine BM-MSC and AD-MSC cultures in DMEM (basal medium) or osteogenic medium on days 7, 14, and 21 *(P < 0.05).

Except for AD-MSCs on days 14 and 21, collagen synthesis was higher in the groups subjected to osteogenic differentiation compared to the undifferentiated groups. On days 14 and 21, BM-MSCs exhibited greater collagen synthesis compared to AD-MSCs under the same culture conditions, and the opposite result was found on day 7 (Figure 4).

**Figure 5:**
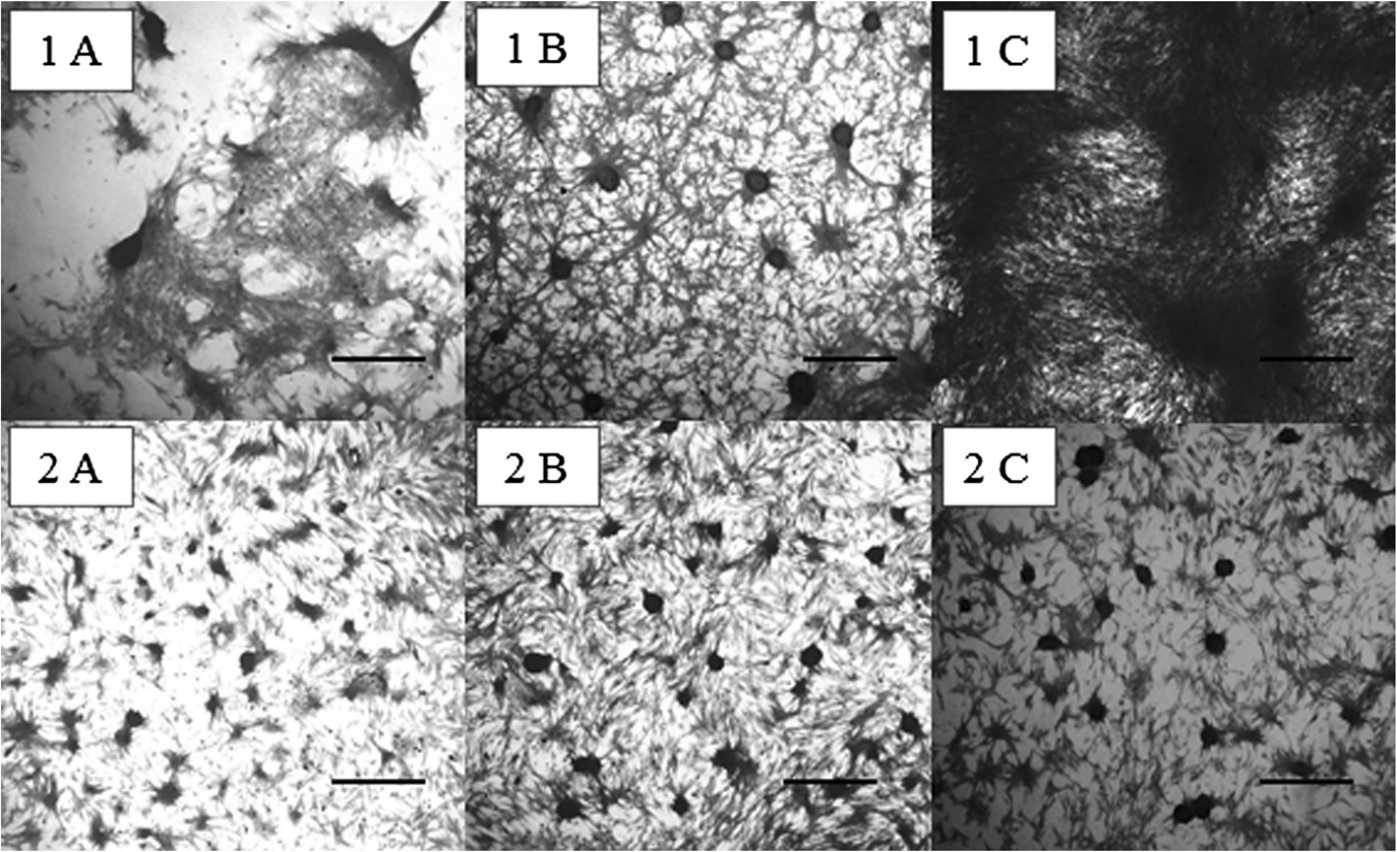
**Culture of mesenchymal stem cells extracted from the bone marrow (BM-MSCs) (1) and adipose tissue (AD-MSCs) (2) of young dogs subjected to osteogenic differentiation at days 7 (A), 14 (B), and 21 (C).** The mineralised nodules were stained by the Von Kossa technique. The mineralised area per field was greater in BM-MSCs (1) compared to AD-MSCs (2) at all of the investigated time-points. Bar = 350 μm.

**Figure 6:**
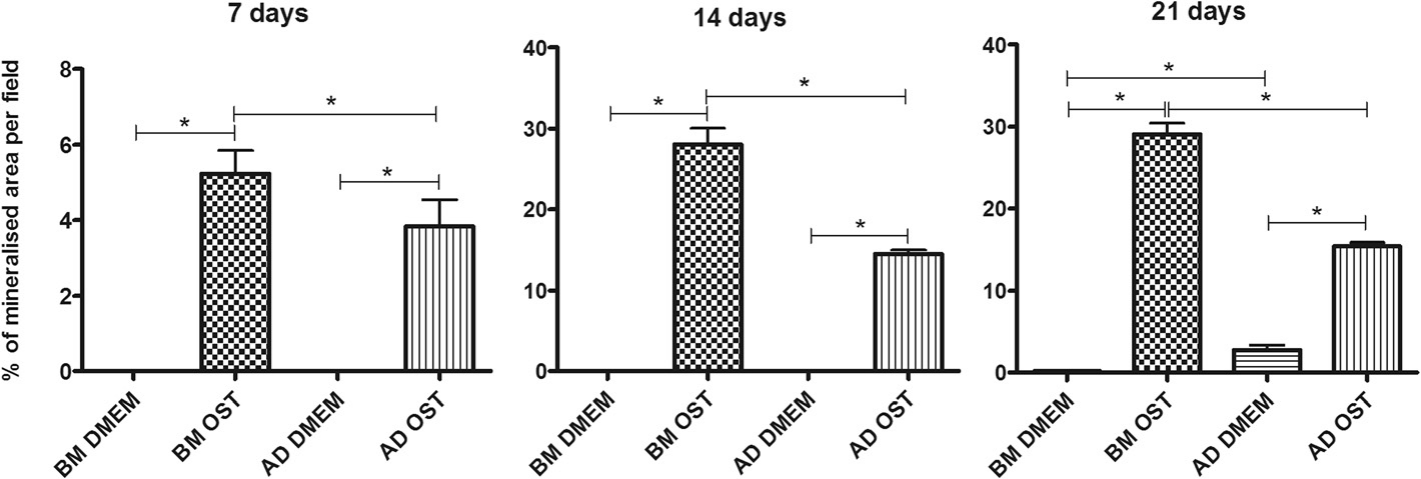
Mean and standard deviation of the percentage of mineralised area per field in canine BM-MSC and AD-MSC cultures in DMEM (basal medium) or osteogenic medium on days 7, 14, and 21 *(P < 0.05).

Both cell types exhibited the morphologic changes typical of osteogenic differentiation on day 7 in osteogenic medium, including nodule formation and the production of mineralised matrix. In the differentiated groups, the percentage of mineralised matrix increased throughout the culture period, while the formation of mineralised matrix was minimal in the undifferentiated groups at less than 3% per field. Relative to the cells subjected to osteogenic differentiation, the percentage of mineralised matrix per field was systematically higher in BM-MSCs compared to AD-MSCs (Figures 5 and 6).

### Gene transcripts of osteogenic differentiation

**Figure 7:**
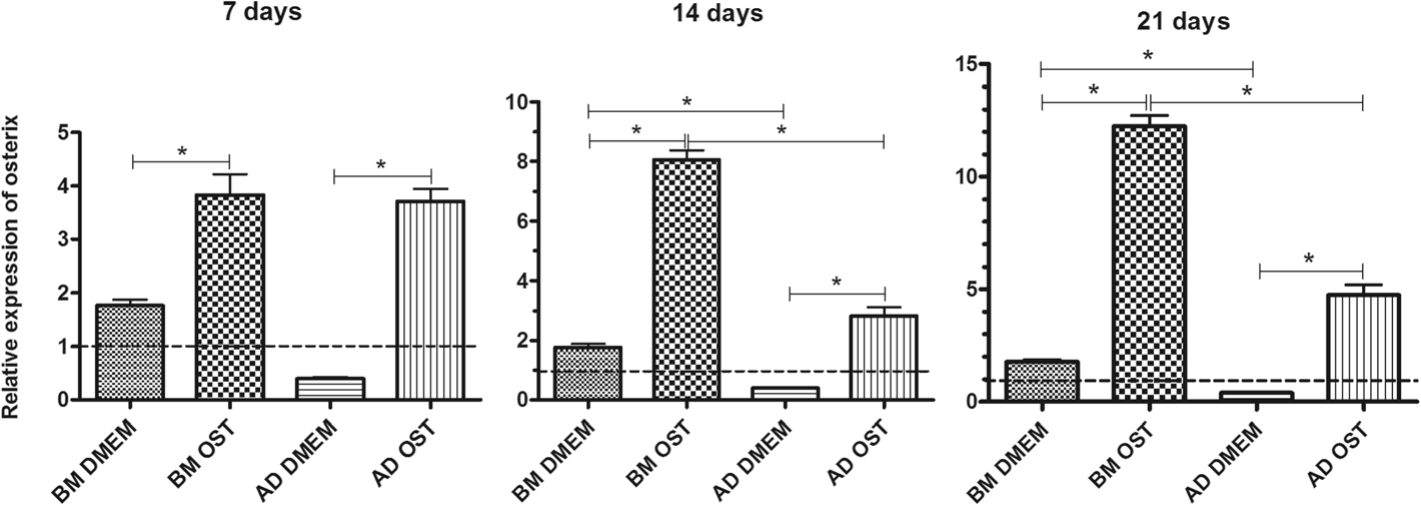
**Mean and standard deviation of relative quantification of osterix gene transcription by qRT-PCR of MSCs in DMEM (basal medium) or osteogenic medium on days 7, 14, and 21.** The data are expressed relative to osteoblasts (dotted line) *(P < 0.05).

Osterix expression was higher in MSCs subjected to osteogenic differentiation compared to those kept in basal medium and those of osteoblasts. On days 14 and 21 of osteogenic differentiation, osterix expression was greater in BM-MSCs compared to AD-MSCs under the same culture conditions, and no difference was found on day 7 (Figure 7).

**Figure 8:**
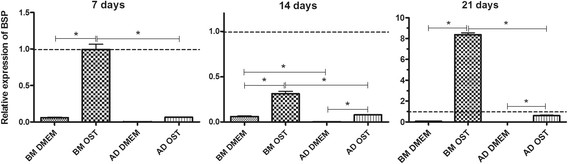
**Mean and standard deviation of bone sialoprotein (BSP) gene transcription of canine BM-MSC and AD-MSC in DMEM (basal medium) or osteogenic medium on days 7, 14, and 21 as measured by qRT-PCR.** The data are expressed relative to osteoblasts (dotted line) *(P < 0.05).

The expression of BSP by MSCs was lower compared to osteoblasts. However, BSP expression by BM-MSCs in osteogenic medium on days 7 and 21 was similar compared to osteoblasts. The expression of BSP by MSCs subjected to osteogenic differentiation was higher than that by MSCs kept in basal medium. Relative to the cells subjected to osteogenic differentiation, the expression of BSP by BM-MSCs was higher compared to AD-MSCs (Figure 8).

**Figure 9:**
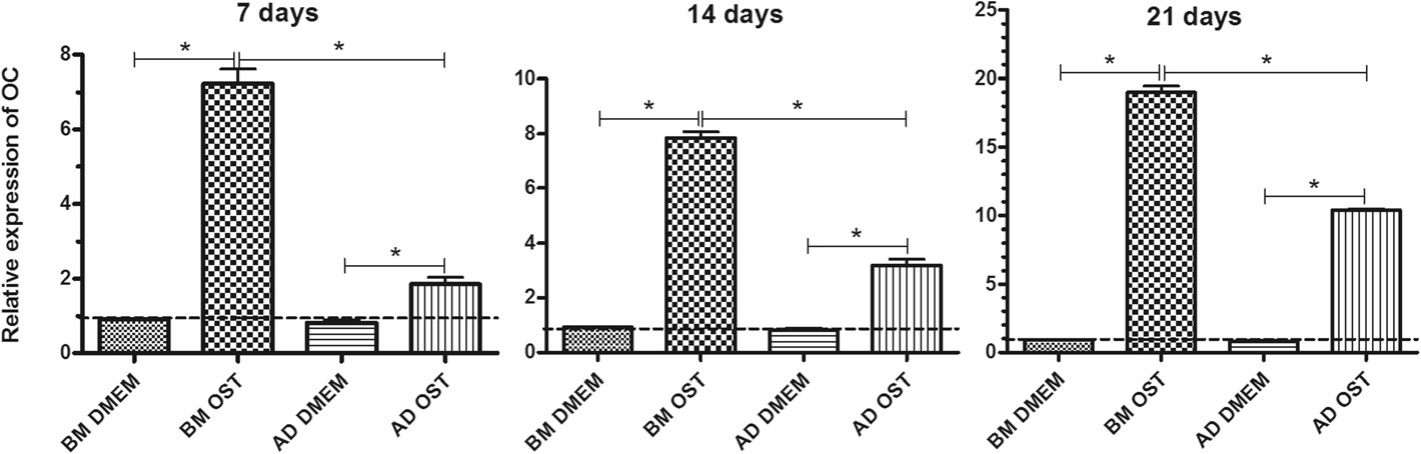
**Mean and standard deviation of osteocalcin (OC) gene transcription of canine BM-MSC and AD-MSC in DMEM (basal medium) or osteogenic medium on days 7, 14, and 21 of osteogenic differentiation as measured by qRT-PCR.** The data are expressed relative to osteoblasts (dotted line) *(P < 0.05).

The expression of OC was systematically higher in the MSCs subjected to osteogenic differentiation compared to the undifferentiated MSCs that were cultured in basal medium and osteoblasts. The expression of OC by BM-MSCs subjected to osteogenic differentiation was systematically higher compared to AD-MSCs under the same culture conditions (Figure 9).

## Discussion

The elongated shape of BM-MSCs and AD-MSCs, as well as the observed phenotypic characteristics of high stem cell marker expression and low haematopoietic cell marker expression, indicated successful isolation of MSCs as per the defined standards of the International Society for Cellular Therapy [13] and reports by other authors [11],[14].

The lower level of MTT conversion into formazan crystals exhibited by the MSCs subjected to osteogenic differentiation was suggestive of lower mitochondrial activity, as found by other authors [1]. This observation may be attributed not only to the lower metabolic activity of differentiated cells compared to undifferentiated MSCs but also to the lower number of cells in the groups subjected to osteogenic differentiation, as seen in the present study. As differentiation progresses, cellular proliferative capacity decreases, and the cell activity shifts to the synthesis of proteins specific to the differentiated cell and the extracellular bone matrix components [15].

The greater MTT conversion found in the BM-MSCs subjected to differentiation indicated greater activity in those cells compared to AD-MSCs under the same culture conditions. This finding represents an advantage in the use of BM-MSCs; the more active the cell, the more extracellular matrix it produces, thus promoting bone regeneration when used for therapeutic purposes.

The gradual and progressive increase of ALP activity during the process of osteogenic differentiation found in both BM-MSCs and AD-MSCs suggested successful osteogenic differentiation [6],[10]. The enzyme ALP is present in the osteoblast membrane, participates in the synthesis and mineralisation of the bone matrix, and is one of the most widely used markers of *in vitro* osteogenic differentiation [1]. Its expression may increase up to 27 times during the process of osteogenic differentiation [16].

The greater collagen synthesis and mineralised matrix per field exhibited by BM-MSCs compared to AD-MSCs subjected to osteogenic differentiation indicate not only improved osteogenic differentiation in the former but also greater capacity for extracellular matrix synthesis under the same culture conditions. Type I collagen is the main component of the organic bone matrix, corresponding to 90% of its composition. It is considered an early marker of osteogenic differentiation and may be expressed by osteoprogenitor cells, pre-osteoblasts, and osteoblasts [17].

The presence of mineralised nodules beginning at day 7 in both MSC types cultured in osteogenic medium confirmed the occurrence of differentiation in these cells. Following osteogenic differentiation, the MSCs become cuboid or polygonal in shape, resembling osteoblasts, and they cluster concentrically, forming mineralised nodules [1]. Such nodules are considered reliable indicators of *in vitro* osteogenic differentiation [18].

The increased osterix expression in the BM-MSC and AD-MSC cultures subjected to differentiation is a relevant indicator of osteogenic differentiation because this transcription factor is indispensable for differentiation. Osterix participates in the transformation of pre-osteoblasts into mature osteoblasts, which is the final step of differentiation [19]. Furthermore, increased osterix expression by MSCs subjected to osteogenic differentiation is considered to be an indicator of differentiation in canines [12].

The greater osterix expression exhibited by BM-MSCs on days 14 and 21 also suggested improved osteogenic differentiation of these cells compared to AD-MSCs under the same culture conditions.

The increased expression of BSP and OC during the differentiation found in the present study is a reflection of the final stage of the osteogenic differentiation of BM-MSCs and AD-MSCs. The non-collagen matrix proteins are considered markers of osteogenic differentiation, and they are closely related to the production and mineralisation of the extracellular matrix [15].

The greater expression of BSP and OC by BM-MSCs found in the present study during the entire process of differentiation gives further proof of their improved osteogenic differentiation compared to AD-MSCs. BSP is a protein that initiates the process of nucleation and deposition of hydroxyapatite in the bone matrix. Increased BSP expression reflects the final stage of differentiation and the onset of matrix mineralisation. It may be expressed by pre-osteoblasts and osteoblasts [16], and increased expression was found in the osteogenic differentiation of AD-MSC [5].

Osteocalcin is a bone-specific glycoprotein that promotes matrix mineralisation. It is expressed by osteoblasts and, therefore, reflects the end of the differentiation and maturation of the bone matrix. It is considered a late and more specific marker of osteogenic differentiation [17],[19]. Increasing levels of OC expression during osteogenic differentiation of BM-MSCs and AD-MSCs has also been reported by other authors [12],[14].

The greater expression of markers of osteogenic differentiation, together with greater collagen synthesis and larger mineralised areas exhibited by BM-MSCs compared to AD-MSCs found in the present study, confirmed the greater osteogenic potential of BM-MSCs in young dogs. Similar results were reported by Im et al. [3], who compared the osteogenic potential of BM-MSCs and AD-MSCs in humans.

## Conclusion

To conclude, BM-MSCs showed greater osteogenic potential compared to AD-MSCs when extracted from young dogs.

## Methods

The present study complied with international standards for animal wellbeing and was approved by the animal experimentation ethics committee (Comitê de Ética em Experimentação Animal - CETEA) of the Federal University of Minas Gerais (Universidade Federal de Minas Gerais – UFMG) (protocol no. 157/2009).

### Harvesting of stem cells and phenotype characterization

Three male mongrel dogs, aged 129 ± 8.29 days and average body weight of 10.17 ± 0.85 kg were used in the study. They were clinically healthy, with negative result for Leishmaniasis and normal total blood count and urinalysis. The animals were given 1 mg/kg xylazine (Calmiun, Agener, São Paulo, Brazil) via the intramuscular (IM) route, 15 mg/kg ketamine (Vetanarcol, Konig, São Paulo, Brazil) IM for anaesthetic induction and preparation for surgery, and 3 mg/kg propofol (Fresofol, Fresenius Kabi, São Paulo, Brazil) via the intravenous route for intubation and maintenance of anaesthesia. Meloxicam (Maxicam, Ouro Fino, São Paulo, Brazil) was given at 0.2 mg/kg IM every 24 hours over 3 days as an analgesic. For harvesting bone marrow, a puncture was performed on the tibial plateau, and 1 mL of bone marrow was collected into 0.5 mL of heparin sodium (Parinex, Hipolabor, São Paulo, Brazil) from each animal, totalling 3 mL. One cm^3^ of adipose tissue was collected from the subcutaneous gluteal area of each animal, totalling 3 cm^3^ of final sample. The bone marrow sample was centrifuged at 1,400 rpm for 10 minutes, the supernatant was discarded, and the pellet was plated and cultured in T75 flasks (Sarstedt, NC, USA) containing low-glucose Dulbecco's modified Eagle's medium – DMEM (Gibco, CA, USA) enriched with gentamycin (60 μg/L), penicillin (100 U/mL), streptomycin (100 μg/mL), amphotericin (25 μg/mL) (PSA, Sigma-Aldrich, USA), and 10% foetal bovine serum (FBS, Soralis, Brazil). The flasks were incubated in a 5% CO_2_ incubator at 37°C, and 48 hours later, the cells were washed with 0.15 M PBS. The adipose tissue sample was washed with 0.15 M PBS and digested in 20 mL of 0.1% w/v collagenase B solution (Roche, Penzberg, Germany). For the purpose of digestion, the sample was fragmented into 2-mm particles and incubated in a 5% CO_2_ incubator at 37°C for 45 minutes. Next, the sample was centrifuged at 1,400 rpm for 10 minutes, the supernatant was discarded, and the pellet was plated and cultured in T75 flasks containing basal culture medium. The flasks were incubated in a 5% CO_2_ incubator at 37°C, and 48 hours later, the cells were washed with 0.15 M PBS. The culture medium was replaced twice per week, and the cells were replated when 80-90% confluence was reached.

The cells were phenotypically characterised for the expression of CD90 (ab22541, Abcam, USA), CD29-PE (ab64629, Abcam, MA, USA), CD45 (ab22514, Abcam, MA, USA), and CD32-PE (ab42902, Abcam, MA, USA) using a secondary antibody (ab6730, Abcam, MA, USA) labelled with FITC for CD90 and CD45, according to the manufacturer’s recommendations. The samples were analysed using a FACScan (Fluorescence-Activated Cell Analyser) flow cytometer and the Cell Quest software. A total of 20,000 events were acquired with the FSC and SSC parameters on the linear scale and the FL1 (for FITC) and FL2 (for PE) parameters on the logarithmic scale. The data were assessed with the WinMDI software using dot plot charts.

### Allocation to experimental groups

After three rounds of replating, MSCs from each source (BM-MSCs and AD-MSCs) were allocated randomly into the following groups: 1) BM-MSCs in osteogenic medium; 2) BM-MSCs in basal medium; 3) AD-MSCs in osteogenic medium; and 4) AD-MSCs in basal medium. The osteogenic medium was composed of the basal culture medium supplemented with 10% FBS (Soralis, São Paulo, Brazil) and enriched with ascorbic acid (50 μg/mL), ß-glycerophosphate (10 mM) (Sigma-Aldrich, MO, USA), and dexamethasone (0.1 μM) (Aché, São Paulo, Brazil). The cells in all four groups were cultured in quadruplicate at 37°C and 5% CO_2_ for 7, 14, and 21 days, and the following parameters were then assessed: conversion of MTT into formazan crystals, alkaline phosphatase (ALP) activity, collagen synthesis, percentage of cells, area of mineralised matrices per field, and relative expression of gene transcripts of osterix (OSX), bone sialoprotein (BSP), and osteocalcin (OC) using real-time reverse transcription polymerase chain reaction (qRT-PCR).

### Conversion of MTT into formazan and alkaline phosphatase activity

To perform the MMT conversion assay, MSCs from each group were cultured separately in 24-well plates containing DMEM (basal medium) or osteogenic medium. At each time-point, the culture medium in each well was replaced by 210 μL of basal medium and 170 μL of MTT (5 mg/mL) (Invitrogen, CA, USA). The plates were incubated in a 5% CO_2_ incubator at 37°C for 2 hours. Formazan crystals were observed under a microscope before the addition of 210 μL of SDS (sodium dodecyl sulphate)-10% HCl, and the samples were left overnight in a 5% CO_2_ incubator at 37°C. Next, a 100 μL aliquot was taken from each well and placed into 96-well plates to be read using a plate reader at 595 nm wavelength, according to Boeloni et al. [9].

To assess ALP activity, MSCs from each group were cultured separately in 24-well plates containing DMEM (basal medium) or osteogenic medium. At each time-point, the cultures were washed with PBS (0.15 M). A total of 200 μL of BCIP/NBT solution (Zymed Laboratories, USA) were added to each well, and the samples were incubated in a 5% CO_2_ incubator at 37°C for 2 hours. The samples were observed under an optical microscope before the addition of 200 μL of SDS (sodium dodecyl sulphate)-10% HCl and were left overnight in a 5% CO_2_ incubator at 37°C. Next, a 100 μL aliquot was taken from each well and placed into 96-well plates to be read using a spectrophotometer at 595 nm wavelength, according to Ocarino et al. [10].

### Collagen synthesis, percentage of cells, and mineralised matrices

To assess collagen synthesis, MSCs from each group were cultured separately in 24-well plates containing DMEM (basal medium) or osteogenic medium. At each time-point, the cultures were washed with PBS (0.15 M). One mL of Bouin fixative was added to each well, and the plates were incubated in a 5% CO_2_ incubator at 37°C for 2 hours and left overnight in a refrigerator at 6°C. At each time-point, the plates were washed four times with reverse osmosis water and dried for subsequent staining with Sirius Red for 30 minutes at room temperature. Excess dye was removed, and the cells were washed three times with 0.01 N HCl solution and dried. Next, 300 μL of 0.5 M NaOH was added, and the plates were incubated for 30 minutes. A 100 μL aliquot was then taken from each well and placed into 96-well plates to be read using a spectrophotometer at 540 nm wavelength, according to Boeloni et al. [20].

To assess the percentage of cells and mineralised matrices per field, MSCs from each group were cultured separately in six-well plates with sterile cover slips (22 × 22 mm) containing DMEM (basal medium) or osteogenic medium. At each time-point, the cultures were washed with 0.15 M PBS, fixed with 70% alcohol for 24 hours, and stained by the Von Kossa method adapted from Prophet et al. [21]. The percentages of cells and mineralised matrices per field were determined from 25 fields using an ocular micrometer comprising a 121-point grid and 4× objective.

### Quantification of gene transcripts of osteogenic differentiation

A commercial primary culture of canine osteoblasts (Abcam, MA, USA) was used as positive control for osteogenic differentiation. The cells were thawed according to the manufacturer’s recommendations and cultured in T75 flasks containing the same media and under the same conditions as BM-MSCs and AD-MSCs. The cultures resulting from the third replating performed after reaching 80-90% confluency were used for the extraction of total mRNA and analysis of osterix, BSP, and OC expressions by qRT-PCR.

To perform relative quantification of the gene transcripts for osterix, BSP, and OC by qRT-PCR, the MSCs of all groups were cultured in quadruplicate in T25 flasks containing DMEM (basal medium) or osteogenic medium. At each time-point, total RNA was extracted from the cultures using TRIzol® (Invitrogen, USA) according to the manufacturer’s instructions. The RNA was solubilised in RNAse-free DEPC water (Invitrogen, USA) and immediately stored at −80°C. RNA concentrations were measured by reading the absorbance at 260/280 nm by spectrophotometry. Reverse transcription was performed using SuperScript™ III Platinum® Two-Step Kit (Invitrogen, USA). The cDNA was synthesised using 1 μg of total RNA in a total volume of 20 μL. Real-time PCR was performed using 2 μg of cDNA, 5 pM of each primer, and 12.5 μL of SYBR® Green reagent (Invitrogen, USA) in a total volume of 25 μL per well using the 7500 Real-Time PCR System (Applied Biosystems, USA). The parameters for amplification were as follows: 50°C for 120 seconds, 95°C for 150 seconds, and 45 cycles of 95°C for 15 seconds and 60°C for 30 seconds. The primers were found in the literature or designed based on the sequence of *Canis familiaris* mRNA (Table 1). Gene expression was calculated using the 2^-∆∆CT^ method, whereby the results of each individual group were compared after normalisation to *Canis familiaris* glyceraldehyde-3-phosphate dehydrogenase (GAPDH) expression. The levels of gene expression measured in the osteoblast cultures were used as positive controls for osteogenic differentiation, and the relative expression of each transcript was calculated based on the expression standard.

**Table 1:** Genes and nucleotide sequences of the primers used in qRT-PCR

**Gene reference**	**Primers (5’ to 3’ nucleotide sequences)**	**Annealing temperature°C**	**Product size pb**
GAPDH [12]	Forward - CCATCTTCCAGGAGCGAGGAT	60	97
	Reverse - TTCTCCATGGTGGTGAAGAC		
Osteocalcin [5]	Forward - GAGGGCAGCGAGGTGGTGAG	62	134
	Reverse - TCAGCCAGCTCGTCACAGTTGG		
Bone sialoprotein (BSP) [12]	Forward – TTGCTCAGCATTTTGGGAAT	60	295
	Reverse - AACGTGGCCGATACTTAAAGAC		
Osterix [5]	Forward – ACGACACTGGGCAAAGCAG	60	285
	Reverse – CATGTCCAGGGAGGTGTAGAC		

### Statistical analysis

Analysis of variance (ANOVA) was performed, and the mean and standard deviation of each variable were calculated. Means were compared with the SNK test using GraphPad InStat3 software (La Jolla, CA, USA). Changes in expression found by qRT-PCR were compared using the SNK test after logarithmic transformation of the data. The significance level was established as p < 0.05 [22].

## Abbreviations

UNIUBE: Universidade de Uberaba

UFMG: Universidade Federal de Minas Gerais

NCT-TCA: Núcleo de Células Tronco e Terapia Celular Animal

BM-MSCs: Mesenchymal Stem Cells extracted from the bone marrow

AD-MSCs: Mesenchymal Stem Cells extracted from the adipose tissue

MSCs: Mesenchymal Stem Cells

qRT-PCR: Real-time reverse transcription polymerase chain reaction

DMEM: Dulbecco’s modified Eagle's medium

MTT: Dimethyl thiazolyl diphenyl tetrazolium

ALP: Alkaline phosphatase

OSX: Osterix

BSP: Sialoprotein

OC: Osteocalcin

CD90: Cluster of Differentiation 90 or THYmocyte differentiation antigen 1

CD29: Cluster of Differentiation 29 or Integrin beta-1

CD45: Cluster of Differentiation 45 or Protein tyrosine phosphatase, receptor type, C

CD34: Cluster of Differentiation 34 or

CETEA: Animal experimentation ethics committee (Comitê de Ética em Experimentação Animal)

FBS: Fetal bovine serum

SDS: (Sodium dodecyl sulphate)

## Competing interests

The authors declare that they have no competing interests.

## Authors’ contributions

EGLA performed the laboratory tests, the statistical analysis and participated in the drafting of the manuscript. RS proposed the idea for the study, and participated in the drafting of the manuscript. JNB and IRR took part in the laboratory tests, the statistical analysis and in the drafting of the manuscript. NMO, HPO and AMG participated in the format and drafting of the manuscript. CMFR coordinated the study, participated in the design and harmonized the drafting of the manuscript. All authors read and approved the final manuscript.
